# Joint Resource Allocation for Multiuser Opportunistic Beamforming Systems with OFDM-NOMA

**DOI:** 10.3390/e23070809

**Published:** 2021-06-25

**Authors:** Wen-Bin Sun, Ming-Liang Tao, Ling Wang, Xin Yang, Rui-Zhe Zhou, Zi-Xiong Yang

**Affiliations:** School of Electronics and Information, Northwestern Polytechnical University, Xi’an 710072, China; sunwenbin@nwpu.edu.cn (W.-B.S.); xinyang@nwpu.edu.cn (X.Y.); zhouruizhe@mail.nwpu.edu.cn (R.-Z.Z.); yangzixiong@mail.nwpu.edu.cn (Z.-X.Y.)

**Keywords:** opportunistic beamforming, multiuser, OFDM, NOMA, spectrum efficiency

## Abstract

Opportunistic beamforming (OBF) is an effective technique to improve the spectrum efficiencies (SEs) of multiple-input-multiple-output (MIMO) systems, which can obtain multiuser diversity gains with both low computation complexity and feedback information. To serve multiple users simultaneously, many multiple-access schemes have been researched in OBF. However, for most of the multiple-access schemes, the SEs are not satisfactory. To further improve the SE, this paper proposes a downlink multiuser OBF system, where both orthogonal frequency division multiplexing (OFDM) and non-orthogonal multiple-access (NOMA) methods are applied. The closed-form expressions of the equivalent channels and SE are derived in frequency selective fading channels. Then, an optimization problem is formulated to maximize the SE, although the optimization problem is non-convex and hard to solve. To obtain the solution, we divide the optimization problem into two suboptimal issues, and then a joint iterative algorithm is applied. In the proposed optimization scheme, the subcarrier mapping ϑ, user pairing $k_{n_{c}}$ and allocated power $P_{k_{n_{c}}}$ are determined to maximize spectrum efficiency (SE) and reduce bit error ratio (BER). According to numerical results, the proposed method achieves approximately 5 dB gain on both SE and BER, compared to the existing beamforming methods with low feedback information. Moreover, the SE of the proposed method is approximately 2 (bps/Hz) higher than sparse code multiple-access (SCMA), when the number of waiting users and the ratio of transmit power to noise variance are respectively 10 and 20 dB. It is indicated that the proposed scheme can achieve high and low BER with the limited feedback and computation complexity, regardless of the transmit power and the number of waiting users.

## 1. Introduction

Multiple-input-multiple-output (MIMO) is one of the key techniques of the next generation of wireless communications, which applies space domain to improve system performance, i.e., capacity and bit error rate (BER) [1,2]. In MIMO systems, precoding is an efficient method to achieve multiuser diversity and multiplexing [3,4]. The conventional precoding schemes contain zero-forcing (ZF) [5,6], regularized channel inversion (RCI) [7,8], block diagonalization (BD) [9,10], etc. For most of the precoding schemes, perfect channel state information (CSI) and complex matrix computation are required, leading to high complexity.

To break the limitation of perfect CSI and reduce the complexity, opportunistic beamforming (OBF) was proposed in [11]. In OBF systems, the received signal-to-noise ratios (SNRs) instead of perfect CSI are required to achieve multiuser diversity, and the precoding weights are randomly generated. The base station (BS) selects the user with the maximum SNR to transmit [12,13]. Moreover, OBF weights introduce large fluctuations into equivalent channels, which results in high multiuser diversity gain and spectrum efficiency (SE) [14].

Due to the low complexity and high SE, many works have been done on OBF. In ref. [15], two transmission mechanisms are proposed for OBF systems, i.e., maximum-capacity-based and fairness-based mechanisms. The closed-form expressions of ergodic capacities and the theoretical bounds of BERs are derived. Ref. [16] analyzes the performances in Rician fading channels and presents the maximum likelihood estimation method of OBF systems. The authors of [17] propose a SNR pre-estimation scheme matching beamforming process, which improves ergodic capacities of OBF systems. In ref. [18], an OBF system based on multiple variable weights is proposed, and the optimal number of OBF weights is derived in a fast-changing independent Rayleigh fading channel. Ref. [19] extends OBF systems from independent fading channels to correlated fading channels, and proposed a low-latency transmission scheme, where the mapping between antenna gain and SNR is pre-loaded at BS for user selection.

The conventional OBF systems only provide single user transmission, then many works began to research the multiple-access schemes of OBF. In [20], multibeam OBF scheme is proposed, and the closed-form expression of ergodic capacity is derived, according to the asymptotic theory of limit distribution of statistics. Then, the authors present the trade-off between multiuser diversity and multiplexing gains. Ref. [21] considers the impact of user location distribution on outage capacity of a multibeam OBF system, and derives the outage capacity, when the locations of users obey Poisson point distributions. Time division multiple-access-based (TDMA-) and space division multiple-access-based (SDMA-) OBF systems are studied in [22], and an algorithm for handoff between TDMA and SDMA schemes is proposed according to the distribution, number and SNRs of users.

The above multibeam OBF systems use orthogonal resources to achieve multiuser multiplexing. To further improve the SE, non-orthogonal patterns are applied. Ref. [23] proposes a non-orthogonal OBF system, where the number of beams is larger than that of transmit antennas. The interferences among the beams is minimized with the non-orthogonal beamforming matrix based on Grassmannian. In [24], a superimposed code is applied to multiplex the data of different users, and the approximate expression of sum rate is derived. The power domain non-orthogonal multiple-access (NOMA) method of multibeam OBF is considered in [25], and then a joint optimal resource allocation algorithm is presented to maximize the sum SE. It is found that multiuser OBF systems can offer the satisfactory quality of service (QoS) to multiple users, simultaneously. In [26], the authors propose a superimposed multiuser shared access (MUSA) scheme based on NOMA method for a massive machine-type communications (mMTC) system. Moreover, the generalized frequency division multiplexing (GFDM) technique is combined with MUSA-NOMA to reduce the latency with high overloading ratio.

However, most of the previous works concentrated on flat fading channels, although few considered frequency selective fading channels. This paper proposes a downlink multiuser OBF system, where OFDM and NOMA schemes are applied to deal with frequency selective fading channels and achieve multiple-access, respectively. Then we derive the closed-form expressions of equivalent channel and SE. Moreover, a joint iterative algorithm is provided to maximum the SE of the proposed system. The main contributions of this paper consist of three aspects.

1. A downlink multiuser opportunistic beamforming with OFDM-NOMA (OBON) system is proposed in frequency selective fading channels, which can obtain both multiuser diversity and multiplexing gains.
2. The improvement of spectrum efficiency caused by OBF weights is taken into consideration. Then, we put forward an optimization problem to maximize the spectrum efficiency with the users’ QoS requirements.
3. Since the optimization problem is non-convex and hard to solve, it is decomposed into two suboptimal problems. According to the analyses of the two suboptimal problems, we propose a joint iterative algorithm is to obtain the solution of the original optimization problem.

The rest of this paper is organized as follows. In Section 2 a multiuser downlink OBON system is introduced. Section 3 formulates an optimization problem to maximize the spectrum efficiency. We propose a joint iterative algorithm to solve the optimization problem, and analyze the convergence and complexity of the algorithm in Section 4. In Section 5, numerical results are provided. Finally, we draw a conclusion in Section 6.

## 2. System Model

Let *x*, x, X be a variable, a vector and a matrix, respectively. $x^{T}$ and $x^{H}$ indicate transpose and conjugate transposes, respectively. ∥·∥ represents the Frobenius norm of a matrix or a vector, and |·| is the absolute value of a variable. C denotes the complex space. E{·} and Var{·} represent expectation and variance, respectively. ⌈x⌉ denotes the minimum integer larger than *x*. The meanings of the notations are listed in Appendix A. During the rest of this paper, we use “NOMA” to present “power-domain NOMA” for simplification.

**Figure 1:**
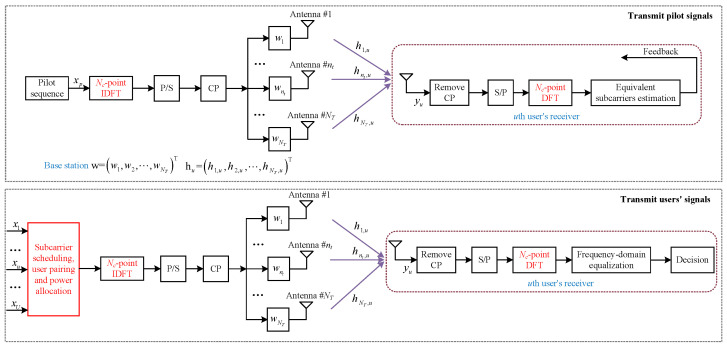
The downlink OBON system, which contains both pilot and user’s signals transmissions.

As it can be seen in Figure 1, a BS and *U* (U≥2) waiting users are involved in an OBON system. The BS has $N_{T}$ $\left(N_{T}\geq 2\right)$ transmit antennas, and each user has only one receive antenna. Assume that the locations of all the users are random. NOMA schemes are applied to multiplex users. Denote the number of subcarriers by $N_{c}$ $\left(N_{c}\geq 1\right)$. The maximum number of the multiplexed users on each subcarrier is K=2, to reduce the complexity of NOMA scheme. Before transmitting users’ signals, pilot signal is sent to evaluate all the subcarriers. To simplify the analyses, the processes of parallel-to-serial, serial-to-parallel, adding and removing cyclic prefix (CP) are ignored.

Pilot vector is denoted by $x_{p}$, which is given by

(1) $$x_{p}={\left[x_{p},x_{p},\cdots ,x_{p}\right]}^{T}\in C^{N_{c}\times 1},$$

where $x_{p}$ represents the element of $x_{p}$, and $x_{p}$ satisfies ${∥x_{p}∥}^{2}=1$. Pilot vector $x_{p}$ is applied to estimate the received SNRs of users on each subcarrier.

To preprocess the transmit signals, a random generated weight vector w is introduced, which is given by

(2) $$w={\left[w_{1},w_{2},\cdots ,w_{n_{t}},\cdots ,w_{N_{T}}\right]}^{T},$$

where $w\in C^{N_{T}\times 1}$ and ${∥w∥}^{2}=1$. $n_{t}$ stands for the label of the transmit antenna and satisfies $N_{T}\geq n_{t}\geq 1$. $w_{n_{t}}$ is the $n_{t}$th element of w, which is achieved as

(3) $$w_{n_{t}}=\sqrt{\alpha_{n_{t}}}e^{j\phi_{n_{t}}},$$

where $\sqrt{\alpha_{n_{t}}}$, $\phi_{n_{t}}$ and *j* represent the amplitude, phase and imaginary number symbol, respectively.

Thus, the transmit signal of pilot signal is

(4) $$s_{p}=w{\bar{x}}_{p},$$

where ${\bar{x}}_{p}$ represents the inverse discrete Fourier transform (IDFT) of pilot signal.

Denote by $h_{n_{t},u}$ the channel coefficient between the $n_{t}$th transmit antenna of the BS and the *u*th (U≥u≥1) user, which is treated as a frequency selective fading channel. Assume that $h_{n_{t},u}$ keeps constant during a bandwidth $B_{sub}$ and coherent time, where $B_{sub}$ is the same-sized bandwidth allocated to each subcarrier. The channel between the BS and the *u*th user can be expressed in a vector form as

(5) $$h_{u}={\left[h_{1,u},h_{2,u},\cdots ,h_{n_{t},u},\cdots ,h_{N_{T},u}\right]}^{T}.$$

The received signal of the *u*th user is

(6) $$y_{p}=h_{u}^{T}s_{p}+z_{u}=h_{u}^{T}w{\bar{x}}_{p}+z_{u}=\sum_{n_{t}=1}^{N_{T}}h_{n_{t},u}w_{n_{t}}{\bar{x}}_{p}+z_{u},$$

where $z_{u}$ represents additive white Gaussian noise (AWGN) vector, and equals to

(7) $$z_{u}=z_{n_{t}}\times {\left[1,1,\cdots ,1\right]}^{T}\in C^{N_{c}\times 1}.$$

$z_{n_{t}}$ is AWGN of the $n_{t}$th transmit antenna with variance $\sigma^{2}$.

Passing a $N_{c}$-point discrete Fourier transform (DFT), the received signal is transformed to

(8) $$\begin{array}{cc} {\tilde{y}}_{p} & =F_{D}\left(\sum_{n_{t}=1}^{N_{T}}h_{n_{t},u}w_{n_{t}}{\bar{x}}_{p}+z_{u}\right)=F_{D}\left(\sum_{n_{t}=1}^{N_{T}}h_{n_{t},u}w_{n_{t}}\frac{1}{N_{c}}F_{I}x_{p}+z_{u}\right)\\ \; & =\sum_{n_{t}=1}^{N_{T}}h_{n_{t},u}w_{n_{t}}\frac{1}{N_{c}}F_{D}F_{I}x_{p}+F_{D}z_{u}=\sum_{n_{t}=1}^{N_{T}}h_{n_{t},u}w_{n_{t}}x_{p}+{\tilde{z}}_{u}, \end{array}$$

where ${\tilde{z}}_{u}$ is also AWGN, due to the isotropy characteristic of AWGN. $F_{D}$ and $F_{I}$ denote DFT and IDFT matrixes, respectively.

Define the equivalent channel by $h_{u}^{\mathrm{equ}}$, which is given by

(9) $$h_{u}^{\mathrm{equ}}\overset{\Delta }{=}\sum_{n_{t}=1}^{N_{T}}h_{n_{t},u}w_{n_{t}}.$$

Combine (8) and (9), we have

(10) $${\tilde{y}}_{p}=h_{u}^{\mathrm{equ}}x_{p}+{\tilde{z}}_{u},$$

According to the known pilot signal, the *u*th user estimates its own equivalent channel $h_{u}^{\mathrm{equ}}$ and then feeds $h_{u}^{\mathrm{equ}}$ back to the BS. It is noted that compared to the conventional preprocessing schemes, the number of feedback bits and the complexity of feedback link in OBON scheme are greatly reduced, since each user is only required to return its equivalent channels $h_{u}^{\mathrm{equ}}$ rather than the complete CSI of all the antennas.

To obtain the channel coefficients of all the subcarriers, $N_{c}$-point DFT is operated for the equivalent channels $h_{u}^{\mathrm{equ}}$, which is achieved as

(11) $${\tilde{h}}_{u}^{\mathrm{equ}}=\left[l_{0,u},l_{1,u},\cdots ,l_{n_{c},u},\cdots ,l_{N_{c}-1,u}\right],$$

where $n_{c}$ is the label of subcarrier and satisfies $\left(N_{c}-1\right)\geq n_{c}\geq 0$. $l_{n_{c},u}$ represents the channel coefficient of the $n_{c}$th subcarrier for the *u*th user.

First, the BS randomly selects K=2 users from *U* waiting users to be multiplexed on each subcarrier, known as $k_{n_{c}}$$\left(K\geq k_{n_{c}}\geq 1\right)$. The mapping between $k_{n_{c}}$ and *u* is defined by $k_{n_{c}}=\kappa \left(u\right)$. Then, a joint resource allocation is applied to determine the optimal user pair, subcarrier schedule and power allocation.

Denote by $P_{k_{n_{c}}}$ the power allocated to the $k_{n_{c}}$th user. Without loss of generality, let $P_{1_{n_{c}}}\geq P_{2_{n_{c}}}$. Thus, the superposition transmit signal on the $n_{c}$th subcarrier is

(12) $$d_{n_{c}}=\sum_{k_{n_{c}}=1}^{K}\sqrt{P_{k_{n_{c}}}}\times x_{k_{n_{c}}},$$

where $x_{k_{n_{c}}}$ represents the desired signal of the $k_{n_{c}}$th user on the $n_{c}$th subcarrier and satisfies ${\left|x_{k_{n_{c}}}x_{k_{n_{c}}}^{*}\right|}^{2}=1$.

Thus, the received signal of the $k_{n_{c}}$th user on the $n_{c}$th subcarrier is given by

(13) $$\begin{array}{cc} y_{k_{n_{c}}} & =l_{n_{c},k_{n_{c}}}d_{n_{c}}+z_{k_{n_{c}}}=l_{n_{c},k_{n_{c}}}\sum_{k_{n_{c}}=1}^{K}\sqrt{P_{k_{n_{c}}}}x_{k_{n_{c}}}+z_{k_{n_{c}}}\\ \; & =l_{n_{c},k_{n_{c}}}\sqrt{P_{k_{n_{c}}}}x_{k_{n_{c}}}+l_{n_{c},k_{n_{c}}}\sum_{k_{n_{c}}^{{}^{\prime }}=1,k_{n_{c}}^{{}^{\prime }}\neq k_{n_{c}}}^{K}\sqrt{P_{k_{n_{c}}^{{}^{\prime }}}}x_{k_{n_{c}}^{{}^{\prime }}}+z_{k_{n_{c}}}, \end{array}$$

where the first, second and third terms are the $k_{n_{c}}$ user’s desired signal, interferences and AWGN, respectively.

To detect the desired signal from the superposition signal $y_{k_{n_{c}}}$, successive interference cancellation (SIC) is applied. The $k_{n_{c}}$th user first decides the signal with the largest power, then removes the influence of the decided signal, and repeat the previous processes until detecting its own desired signal.

Therefore, the signal-to-interference-plus-noise ratio (SINR) of the $k_{n_{c}}$th user can be expressed as

(14) $$\gamma_{k_{n_{c}}}=\frac{P_{k_{n_{c}}}{\left|l_{n_{c},k_{n_{c}}}\right|}^{2}}{\sum_{k_{n_{c}}^{{}^{\prime }}=k_{n_{c}}+1}^{K}P_{k_{n_{c}}^{{}^{\prime }}}{\left|l_{n_{c},k_{n_{c}}}\right|}^{2}+\sigma^{2}}.$$

The rate of the $k_{n_{c}}$th user on the $n_{c}$th subcarrier is

(15) $$R_{k_{n_{c}}}=B_{sub}\log_{2}\left(1+\frac{P_{k_{n_{c}}}{\left|l_{n_{c},k_{n_{c}}}\right|}^{2}}{\sum_{k_{n_{c}}^{{}^{\prime }}=k_{n_{c}}+1}^{K}P_{k_{n_{c}}^{{}^{\prime }}}{\left|l_{n_{c},k_{n_{c}}}\right|}^{2}+\sigma^{2}}\right).$$

Therefore, the spectrum efficiency (SE) of OBON can be calculated as

(16) $$\begin{array}{cc} E_{\mathrm{total}} & =\frac{\sum_{n_{c}=0}^{N_{c}-1}\sum_{k_{n_{c}}=1}^{K}B_{sub}\log_{2}\left(1+\frac{P_{k_{n_{c}}}{\left|l_{n_{c},k_{n_{c}}}\right|}^{2}}{\sum_{k_{n_{c}}^{{}^{\prime }}=k_{n_{c}}+1}^{K}P_{k_{n_{c}}^{{}^{\prime }}}{\left|l_{n_{c},k_{n_{c}}}\right|}^{2}+\sigma^{2}}\right)}{N_{c}B_{sub}}\\ \; & =\frac{1}{N_{c}}\sum_{n_{c}=0}^{N_{c}-1}\sum_{k_{n_{c}}=1}^{K}\log_{2}\left(1+\frac{P_{k_{n_{c}}}{\left|l_{n_{c},k_{n_{c}}}\right|}^{2}}{\sum_{k_{n_{c}}^{{}^{\prime }}=k_{n_{c}}+1}^{K}P_{k_{n_{c}}^{{}^{\prime }}}{\left|l_{n_{c},k_{n_{c}}}\right|}^{2}+\sigma^{2}}\right). \end{array}$$

## 3. An Optimization Problem to Maximize SE

Based on the aforementioned analyses, an optimization problem is formulated to maximize the SE of OBON system in this section. The objective function and constraints are introduced one-by-one.

### 3.1. Objective Function

To achieve the maximum SE of OBON system, the objective function is founded as

(17) $$\max_{\vartheta ,k_{n_{c}},P_{k_{n_{c}}}}E_{\mathrm{total}}=\max_{\vartheta ,k_{n_{c}},P_{k_{n_{c}}}}\frac{1}{N_{c}}\sum_{n_{c}=0}^{N_{c}-1}\sum_{k_{n_{c}}=1}^{K}\log_{2}\left(1+\frac{P_{k_{n_{c}}}{\left|l_{n_{c},k_{n_{c}}}\right|}^{2}}{\sum_{k_{n_{c}}^{{}^{\prime }}=k_{n_{c}}+1}^{K}P_{k_{n_{c}}^{{}^{\prime }}}{\left|l_{n_{c},k_{n_{c}}}\right|}^{2}+\sigma^{2}}\right).$$

Due to both SIC and NOMA, objective function (17) can be rewritten as

(18) $$\max_{\vartheta ,k_{n_{c}},P_{k_{n_{c}}}}\sum_{n_{c}=0}^{N_{c}-1}\left[\log_{2}\left(1+\frac{P_{1_{n_{c}}}{\left|l_{n_{c},k_{n_{c}}}\right|}^{2}}{P_{2_{n_{c}}}{\left|l_{n_{c},k_{n_{c}}}\right|}^{2}+\sigma^{2}}\right)+\log_{2}\left(1+\frac{P_{2_{n_{c}}}{\left|l_{n_{c},k_{n_{c}}}\right|}^{2}}{\sigma^{2}}\right)\right].$$

### 3.2. Constraints

NOMA power constraint: Based on the assumption $P_{1_{n_{c}}}\geq P_{2_{n_{c}}}$ and SIC power difference requirement, the power of users satisfies

(19) $$C1:P_{1_{n_{c}}}-P_{2_{n_{c}}}\geq P_{\Delta },$$

where $P_{\Delta }$$\left(0\leq P_{\Delta }<P_{\mathrm{total}}\right)$ denotes the minimum power difference between the users to distinguish users’ signals.

Total power constraint: To guarantee a stable transmit power, we set that the total power of each subcarrier equals a constant, as

(20) $$C2:P_{1_{n_{c}}}+P_{2_{n_{c}}}=P_{\mathrm{total}},$$

where $P_{\mathrm{total}}$ is the total power of each subcarrier.

Users’ quality of service (QoS) constraint: To ensure the QoS of the selected users, we assume that the SE of the $k_{n_{c}}$th user on the $n_{c}$th subcarrier is larger than a required minimum SE, as

(21) $$C3:\frac{R_{k_{n_{c}}}}{B_{sub}}\geq e_{k_{n_{c}}}.$$

User pairing constraint: The BS selects K=2 users from *U* waiting users to pair, thus, all the user pair combinations can be represented as a set, given as

(22) $$U_{s}=\left\{\left(1,2\right),\ldots ,\left(1,U\right),\left(2,1\right),\ldots ,\left(2,U\right),\ldots ,\left(u_{1},u_{2}\right),\ldots ,\left(U,1\right),\ldots ,\left(U,U-1\right)\right\},$$

where $\left(u_{1},u_{2}\right)$ represents a user pair combination, $1\leq u_{1},u_{2}\leq U$ and $u_{1}\neq u_{2}$.

According to (22), user pairing constraint is given by

(23) $$C4:\left(1_{n_{c}},2_{n_{c}}\right)\in U_{s}.$$

### 3.3. Optimization Problem

According to the objective function and constraints, the optimization problem to maximize the SE of OBON is formulated as

(24) $$\begin{array}{cc} \; & \max_{\vartheta ,k_{n_{c}},P_{k_{n_{c}}}}D=\sum_{n_{c}=0}^{N_{c}-1}\left[\log_{2}\left(1+\frac{P_{1_{n_{c}}}{\left|l_{n_{c},k_{n_{c}}}\right|}^{2}}{P_{2_{n_{c}}}{\left|l_{n_{c},k_{n_{c}}}\right|}^{2}+\sigma^{2}}\right)+\log_{2}\left(1+\frac{P_{2_{n_{c}}}{\left|l_{n_{c},k_{n_{c}}}\right|}^{2}}{\sigma^{2}}\right)\right],\\ \; & s.t.\;C1-C4. \end{array}$$

It is found the optimization problem (24) is a non-convex problem and hard to solve, due to the following reasons:

(1) The objective function *D* contains non-polynomial elements, leading to the non-convexity of the objective function.
(2) Both C3 and C4 constraints are non-convex.

## 4. Problem Solution

To solve the proposed optimization problem (24), we first divide it into two suboptimal problems, then solve the suboptimal problems one-by-one, and finally apply a joint iterative algorithm to obtain the optimal solution.

### 4.1. Power Allocation

Considering the given user pairing and subcarrier scheduling scenario, the optimization problem (24) degenerates into a power allocation issue, which is given by

(25) $$\begin{array}{cc} \; & \max_{P_{k_{n_{c}}}}D_{1}=\log_{2}\left(1+\frac{P_{1_{n_{c}}}{\left|l_{n_{c},1_{n_{c}}}\right|}^{2}}{P_{2_{n_{c}}}{\left|l_{n_{c},1_{n_{c}}}\right|}^{2}+\sigma^{2}}\right)+\log_{2}\left(1+\frac{P_{2_{n_{c}}}{\left|l_{n_{c},2_{n_{c}}}\right|}^{2}}{\sigma^{2}}\right),\\ \; & s.t.\;C1-C3. \end{array}$$

Substitute $P_{1_{n_{c}}}$ and $P_{2_{n_{c}}}$ by $\left(1-\zeta \right)P_{\mathrm{total}}$ and $\zeta P_{\mathrm{total}}$, respectively. ζ is power allocation weight, and satisfies 0≤ζ≤1. According to the characteristics of logarithmic function, the power allocation issue (25) can be transformed as

(26) $$\begin{array}{cc} \; & \max_{\zeta }{\tilde{D}}_{1}=\left(\frac{P_{\mathrm{total}}{\left|l_{n_{c},1_{n_{c}}}\right|}^{2}+\sigma^{2}}{\zeta P_{\mathrm{total}}{\left|l_{n_{c},1_{n_{c}}}\right|}^{2}+\sigma^{2}}\right)\times \left(\frac{\zeta P_{\mathrm{total}}{\left|l_{n_{c},2_{n_{c}}}\right|}^{2}+\sigma^{2}}{\sigma^{2}}\right),\\ \; & s.t.\;C5:0\leq \zeta \leq \frac{P_{\mathrm{total}}-P_{\Delta }}{2P_{\mathrm{total}}},\\ \; & s.t.\;C6:\log_{2}\left(\frac{P_{\mathrm{total}}{\left|l_{n_{c},1_{n_{c}}}\right|}^{2}+\sigma^{2}}{\zeta P_{\mathrm{total}}{\left|l_{n_{c},1_{n_{c}}}\right|}^{2}+\sigma^{2}}\right)\geq e_{1_{n_{c}}},\\ \; & s.t.\;C7:\log_{2}\left(\frac{\zeta P_{\mathrm{total}}{\left|l_{n_{c},2_{n_{c}}}\right|}^{2}+\sigma^{2}}{\sigma^{2}}\right)\geq e_{2_{n_{c}}}. \end{array}$$

For mathematical convenience, set $\frac{P_{\mathrm{total}}}{\sigma^{2}}=\vartheta$. The power allocation issue can be further simplified as

(27) $$\begin{array}{cc} \; & \max_{\zeta }{\bar{D}}_{1}=\left(\frac{\vartheta {\left|l_{n_{c},1_{n_{c}}}\right|}^{2}+1}{\vartheta {\left|l_{n_{c},1_{n_{c}}}\right|}^{2}\zeta +1}\right)\times \left(\vartheta {\left|l_{n_{c},2_{n_{c}}}\right|}^{2}\zeta +1\right),\\ s.t. & \;C8:0\leq \zeta \leq \frac{P_{\mathrm{total}}-P_{\Delta }}{2P_{\mathrm{total}}},\\ \; & \;C9:\zeta \leq \frac{\frac{\vartheta {\left|l_{n_{c},1_{n_{c}}}\right|}^{2}+1}{2^{e_{1_{n_{c}}}}}-1}{\vartheta {\left|l_{n_{c},1_{n_{c}}}\right|}^{2}}\\ \; & \;C10:\zeta \geq \frac{2^{e_{2_{n_{c}}}}-1}{\vartheta {\left|l_{n_{c},2_{n_{c}}}\right|}^{2}}. \end{array}$$

The derivative of ${\bar{D}}_{1}$ to ζ is

(28) $$\begin{array}{cc} \frac{d{\bar{D}}_{1}}{d\zeta } & =\frac{\vartheta \left(\vartheta {\left|l_{n_{c},1_{n_{c}}}\right|}^{2}+1\right){\left|l_{n_{c},2_{n_{c}}}\right|}^{2}}{\vartheta {\left|l_{n_{c},1_{n_{c}}}\right|}^{2}\zeta +1}-\frac{\vartheta {\left|l_{n_{c},1_{n_{c}}}\right|}^{2}\left(\vartheta {\left|l_{n_{c},1_{n_{c}}}\right|}^{2}+1\right)\left(\vartheta {\left|l_{n_{c},2_{n_{c}}}\right|}^{2}\zeta +1\right)}{{\left(\vartheta {\left|l_{n_{c},1_{n_{c}}}\right|}^{2}\zeta +1\right)}^{2}}\\ \; & =\frac{\vartheta \left(\vartheta {\left|l_{n_{c},1_{n_{c}}}\right|}^{2}+1\right)\left({\left|l_{n_{c},2_{n_{c}}}\right|}^{2}-{\left|l_{n_{c},1_{n_{c}}}\right|}^{2}\right)}{{\left(\vartheta {\left|l_{n_{c},1_{n_{c}}}\right|}^{2}\zeta +1\right)}^{2}}. \end{array}$$

It is indicated that ${\bar{D}}_{1}$ is monotonous with respect to ζ, and the monotonicity of is determined by $\left({\left|l_{n_{c},2_{n_{c}}}\right|}^{2}-{\left|l_{n_{c},1_{n_{c}}}\right|}^{2}\right)$. Thus, the optimal $\zeta_{opt}$ can be achieved by comparing the values of ${\bar{D}}_{1}$ at the endpoints of the feasible interval.

According to C8–C10, the feasible interval is given by

(29) $$\begin{array}{cc} \; & G=\left[G_{\zeta ,\mathrm{left}},G_{\zeta ,\mathrm{right}}\right]\\ \;\; & =\left[\max\left\{0,\frac{2^{e_{2_{n_{c}}}}-1}{\vartheta {\left|l_{n_{c},2_{n_{c}}}\right|}^{2}}\right\},\min\left\{\frac{P_{\mathrm{total}}-P_{\Delta }}{2P_{\mathrm{total}}},\frac{\frac{\vartheta {\left|l_{n_{c},1_{n_{c}}}\right|}^{2}+1}{2^{e_{1_{n_{c}}}}}-1}{\vartheta {\left|l_{n_{c},1_{n_{c}}}\right|}^{2}}\right\}\right]. \end{array}$$

The optimal $\zeta_{opt}$ is given by

(30) $$\begin{array}{c} \zeta_{opt}=\underset{G_{\zeta ,\mathrm{left}},G_{\zeta ,\mathrm{right}}}{\arg}\max{\bar{D}}_{1}. \end{array}$$

Therefore, the power allocation result is $P_{1_{n_{c}}}=\left(1-\zeta_{opt}\right)P_{\mathrm{total}}$ and $P_{2_{n_{c}}}=\zeta_{opt}P_{\mathrm{total}}$.

### 4.2. Subcarrier Scheduling and User Pairing

In this subsection, we discuss subcarrier scheduling and user pairing scheme, when the power allocation result is fixed. The optimization problem (24) can be rewritten as

(31) $$\begin{array}{cc} \; & \max_{\vartheta ,k_{n_{c}}}D_{2}=\sum_{n_{c}=0}^{N_{c}-1}\left[\log_{2}\left(1+\frac{P_{1_{n_{c}}}{\left|l_{n_{c},k_{n_{c}}}\right|}^{2}}{P_{2_{n_{c}}}{\left|l_{n_{c},k_{n_{c}}}\right|}^{2}+\sigma^{2}}\right)+\log_{2}\left(1+\frac{P_{2_{n_{c}}}{\left|l_{n_{c},k_{n_{c}}}\right|}^{2}}{\sigma^{2}}\right)\right],\\ \; & s.t.\;C4:\left(1_{n_{c}},2_{n_{c}}\right)\in U_{s}. \end{array}$$

Since the number of subcarriers $N_{c}$, the dimensions of ϑ and the elements of $U_{s}$ are finite, an exhaustive searching algorithm can be applied to achieve the optimal solution of (31), as shown in Algorithm 1.
**Algorithm 1:** Subcarrier scheduling and user pairing scheme.1:**Input:**$N_{c}$, *U*, ${\left|l_{n_{c},k_{n_{c}}}\right|}^{2}$, $P_{1,n_{c}}$ and $P_{2,n_{c}}$.2:**Output:** Subcarrier mapping ϑ and user pairing $k_{n_{c}}$.3:Initialize: $n_{c}=0$ and i=1, where *i* denotes the *i*th element of $U_{s}$;4:**while**$n_{c}\in \left[0,N_{c}-1\right]$**do**5: **while**
i∈[1,U·(U−1)]
**do**6:  Select the *i*th element of $U_{s}$;7:  Determine the $1_{n_{c}}$th and $2_{n_{c}}$th users;8:  Calculate $E_{\mathrm{total}}$ as (16);9:  Record $E_{\mathrm{total}}$, subcarrier scheduling ϑ and user pairing $k_{n_{c}}$;10:  i=i+1;11: **end while**12: i=1;13: $n_{c}=n_{c}+1$;14:**end while**15:Search the maximum $E_{\mathrm{total}}$ and corresponding subcarrier scheduling ϑ and user pairing $k_{n_{c}}$.

According to Algorithm 1, the optimal subcarrier scheduling $k_{n_{c}}$ and user pairing ϑ scheme is determined.

### 4.3. A Joint Iterative Algorithm

To take power allocation, subcarrier scheduling and user pairing into consideration simultaneously, a joint iterative algorithm is proposed, named by Algorithm 2.
**Algorithm 2:** A joint iterative algorithm.1:**Input:**$N_{c}$, *U*, $P_{\mathrm{total}}$ and ${\left|l_{n_{c},k_{n_{c}}}\right|}^{2}$.2:**Output:** Power allocation result $P_{1,n_{c}}$, $P_{2,n_{c}}$, subcarrier mapping ϑ and user pairing $k_{n_{c}}$.3:Initialize: $P_{1,n_{c}}=P_{\mathrm{total}}$, $P_{2,n_{c}}=0$, $n_{c}=0$ and i=1;4:**while**$n_{c}\in \left[0,N_{c}-1\right]$**do**5: **while**
i∈[1,U·(U−1)]
**do**6:  Schedule subcarriers and users based on (31);7:  Allocate power to the paired users according to (25);8:  Calculate $E_{\mathrm{total}}$ with power allocation, subcarrier scheduling and user pairing result;9:  Record $E_{\mathrm{total}}$, power allocation $P_{1,n_{c}}$, $P_{2,n_{c}}$, subcarrier scheduling ϑ and user pairing $k_{n_{c}}$;10:  i=i+1;11: **end while**12: i=1;13: $n_{c}=n_{c}+1$;14:**end while**15:Search the maximum $E_{total}$ and corresponding power allocation $P_{1,n_{c}}$, $P_{2,n_{c}}$, subcarrier scheduling ϑ and user pairing $k_{n_{c}}$.

Algorithm 2 mainly contains three steps, as:

Step 1: Initialization: Set the number of waiting users *U*, total transmit power $P_{\mathrm{total}}$, the number of subcarriers $N_{c}$ and the equivalent channels ${\left|l_{n_{c},k_{n_{c}}}\right|}^{2}$.

Step 2: Three-layer iterations: A three-layer iterative algorithm is user to find the optimal solution.

(1) Outer-layer: $n_{c}$ is from 0 to $\left(N_{c}-1\right)$, which decides the subcarrier scheduling.
(2) Adjacent inner-layer: Search *i* from 1 to U·(U−1) for user pairing.
(3) Inner-layer: According to the given subcarrier scheduling and user pairing scheme, we allocate $P_{1,n_{c}}$ and $P_{2,n_{c}}$ to the selected users, according to (25).

Calculation: Evaluate the SE of OBON system $E_{\mathrm{total}}$ with (16).

Record processing: Store the SE $E_{\mathrm{total}}$, corresponding $P_{1,n_{c}}$, $P_{2,n_{c}}$, ϑ and $k_{n_{c}}$.

Step 3: Output: Output the maximum $E_{\mathrm{total}}$, corresponding $P_{1,n_{c}}$, $P_{2,n_{c}}$, ϑ and $k_{n_{c}}$.

All the parameters, which include power allocation result $P_{1,n_{c}}$, $P_{2,n_{c}}$, subcarrier scheduling $k_{n_{c}}$ and user pairing ϑ scheme, are decided through the proposed joint iterative algorithm. Then the signals of users are superimposed and transmitted together.

### 4.4. Convergence and Complexity

Convergence: Since the proposed joint iterative algorithm includes two suboptimal respects, i.e., power allocation (25) and subcarrier scheduling and user pairing (31) issues, the convergence of Algorithm 2 is determined by both the issues. For power allocation issue, G≠∅ and (30) has a solution, thus the power allocation scheme is convergent. The number of exhaustive searches is finite, which leads to the convergence of subcarrier scheduling and user pairing problem. Considering both the convergences of suboptimal issues, the proposed Algorithm 2 is convergent.

Complexity: As with the analyses of convergence, the complexity of Algorithm 2 can be achieved through the complexities of the two suboptimal issues. The complexity of power allocation is $O\left(\lambda_{p}\right)$, where $\lambda_{p}$ represents the total complexity to complete once derivation (28) and numerical comparison (29). The complexity of subcarrier scheduling and user pairing issue is determined by the number of exhaustive searches and equals to $O\left(N_{c}\times U\times \left(U-1\right)\right)$. Moreover, the computational complexity to generate OBF weight is $O(N_{T}\times U)$. Here, the complexity of Algorithm 2 is expressed as $O(\lambda_{p}\times N_{c}\times N_{T}\times U^{3})$.

Comparison with other existing schemes: There are three kinds of traditional beamforming schemes to deal with the limited feedback information, i.e., Grassmannian subspace packing (GSP), genetic algorithm (GA) and vector quantization (VQ). According to [27,28,29], the computational complexities to obtain beamforming weights of GSP, GA and VQ are $O(N_{T}^{2}\times U^{2}\times L_{\mathrm{gsp}})$, $O(N_{T}^{2}\times U^{2}\times I_{\mathrm{ga}})$ and $O(N_{T}^{2}\times U)$, respectively. $L_{\mathrm{gsp}}$ and $I_{\mathrm{ga}}$ present the dimension of subspace and the number of iterations, respectively. Therefore, the total computational complexities of GSP, GA and VQ are $O(\lambda_{p}\times N_{c}\times N_{T}^{2}\times U^{4}\times L_{\mathrm{gsp}})$, $O(\lambda_{p}\times N_{c}\times N_{T}^{2}\times U^{4}\times I_{\mathrm{ga}})$ and $O(\lambda_{p}\times N_{c}\times N_{T}^{2}\times U^{3})$, respectively. Moreover, an ideal CSI scenario is considered, where coherent beamforming (CBF) scheme [30] is applied to transmit signals. Based on [31], the computational complexity of CBF is $O(\lambda_{p}\times N_{c}\times N_{T}^{3}\times U^{5})$. Comparing the proposed scheme with GSP, GA, VQ and CBF schemes, it is found that the proposed scheme achieves the lowest computational complexity.

### 4.5. Low-Complexity Subcarrier Scheduling and User Pairing Algorithm

An exhaustive traversal is applied in Algorithm 1 to obtain both the optimal user pairing and subcarrier schedule. When the numbers of waiting users and subcarriers are both small, the complexity is tolerable, and the optimal solution can be achieved simply. However, when the numbers of waiting users and subcarriers are significantly boosted, a low-complexity algorithm is required, as shown in Algorithm 3. Compared to Algorithm 1, bipartite graph and distributed queue are applied to simplify the complexity.
**Algorithm 3:** Low-complexity subcarrier scheduling and user pairing scheme.1:**Input:**$N_{c}$, *U*, ${\left|l_{n_{c},k_{n_{c}}}\right|}^{2}$, $P_{1,n_{c}}$ and $P_{2,n_{c}}$.2:**Output:** Subcarrier scheduling $M_{\mathrm{low}}$ and user pairing $k_{n_{c}}$.3:Initialize: $M_{\mathrm{low}}=\emptyset$, $i_{1}=1$ and ς=1, where $M_{\mathrm{low}}$, *i* and ς denote the subcarrier mapping, *i*th element of $U_{s}$ and ςth user pairing result, respectively;4:Arrange the elements of $U_{s}$ in descending order, known as $\bar{U_{s}}$;5:**while**$i\in \left[1,\left\lceil \frac{U}{2}\right\rceil \right]$**do**6: Denote the subset of the use pairing as $\bar{U_{s}^{\mathrm{sub}}}$;7: **if**
$i+\left\lceil \frac{U}{2}\right\rceil =U$
**then**8:  $\bar{U_{s}^{\mathrm{sub}}}=\left[\left(\bar{U_{s}}\left(i\right),\bar{U_{s}}\left(i+\left\lceil \frac{U}{2}\right\rceil \right)\right),\left(\bar{U_{s}}\left(i+\left\lceil \frac{U}{2}\right\rceil \right),\bar{U_{s}}\left(i\right)\right)\right]$;9: **else**10:  $\bar{U_{s}^{\mathrm{sub}}}=\left[\left(\bar{U_{s}}\left(i\right),\bar{U_{s}}\left(i\right)\right),\left(\bar{U_{s}}\left(i\right),\bar{U_{s}}\left(i\right)\right)\right]$;11: **end if**12: **while**
ς∈[1,2]
**do**13:  Select the ςth element of $\bar{U_{s}^{\mathrm{sub}}}$;14:  Determine the 1th and 2th users;15:  Set $C=\left\{\tilde{n_{c}}|\tilde{n_{c}}\in M_{\mathrm{low}}\right\}$, $\tilde{n_{c}}$ is the unsaturated points;16:  **if**
C≠∅
**then**17:   Find a new augmented branch χ;18:   $M_{\mathrm{low}}=M_{\mathrm{low}}⨁\chi$;19:  **else**20:   Calculate $E_{\mathrm{total}}$ as (16);21:   Record $E_{\mathrm{total}}$, subcarrier scheduling $M_{\mathrm{low}}$ and user pairing $k_{n_{c}}$;22:   ς=ς+1;23:  **end if**24:  i=i+1;25: **end while**26: i=1;27:**end while**28:Search the maximum $E_{\mathrm{total}}$ and corresponding subcarrier scheduling $M_{\mathrm{low}}$ and user pairing $k_{n_{c}}$.

## 5. Numerical Results

Numerical results are provided in this section, where we assume the channel coefficients of subcarriers satisfy circular symmetric complex Gaussian random distributions with zero mean and unit variance CN(0,1). During this section, binary phase shift keying (BPSK) is used. The details of simulation parameters are listed in Table 1.

**Figure 2:**
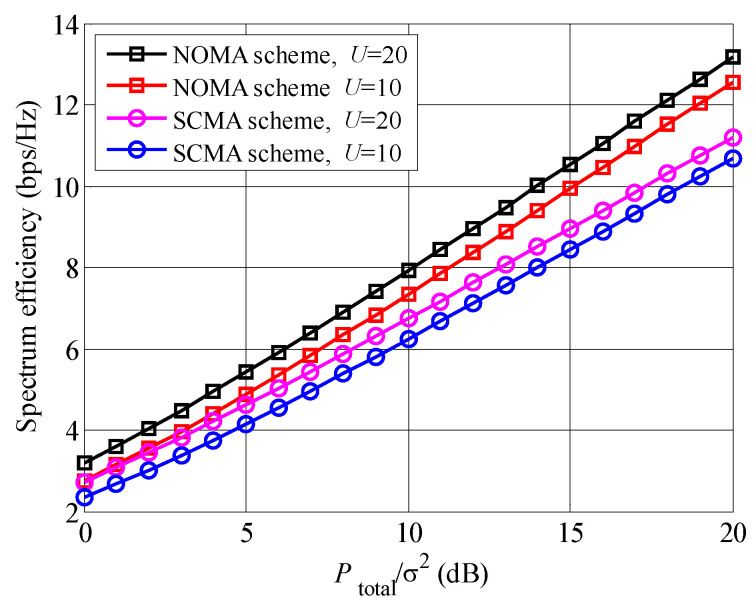
Comparison between different non-orthogonal multiplexing schemes.

The spectrum efficiencies of different non-orthogonal multiplexing schemes with the increased $\frac{P_{\mathrm{total}}}{\sigma^{2}}$ are indicated in Figure 2. It is seen that the spectrum efficiency of the proposed NOMA scheme is larger than that of SCMA scheme. For example, when $\frac{P_{\mathrm{total}}}{\sigma^{2}}=20$ dB and U=20, the spectrum efficiency of NOMA schemes is approximately 13 bps/Hz, although the SCMA scheme achieves approximately 11 bps/Hz. The reason is that resource multiplexing rate of NOMA scheme is higher than that of SCMA scheme. Moreover, comparing the curves between the different *U*, we can draw that the curves of U=20 are higher than the curves of U=10, no matter of the non-orthogonal multiplexing schemes. The larger number of waiting users *U* causes larger equivalent channel $h_{u}^{\mathrm{equ}}$, which leads to higher spectrum efficiency.

**Figure 3:**
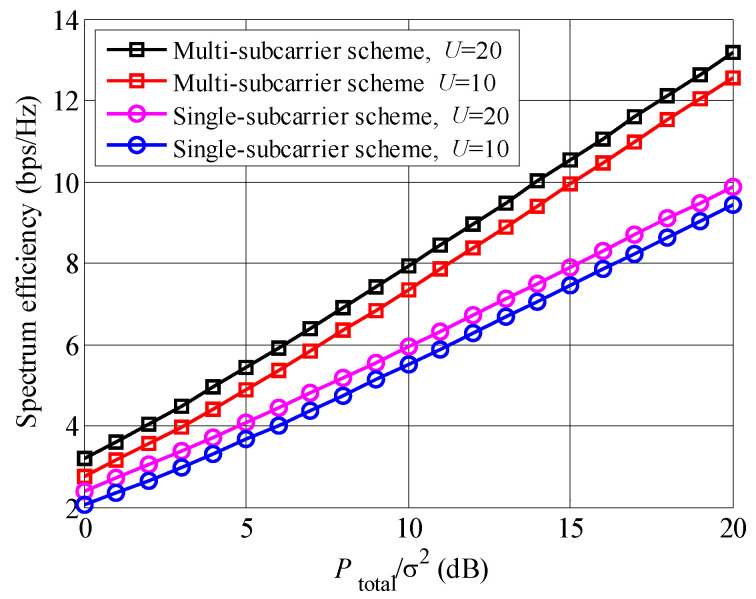
Comparison between multi-subcarrier and single-subcarrier schemes.

Figure 3 shows the comparison between multi-subcarrier and single-subcarrier schemes in the proposed system. It is found that multi-subcarrier scheme achieves higher spectrum efficiency than single-subcarrier. Since the channel coefficients of the subcarriers are different, multi-subcarrier scheme can adaptively adjust the power and user pair on each subcarrier. Moreover, the deep fading point in the frequency band is avoided in multi-subcarrier scheme, which results in higher system performance.

**Figure 4:**
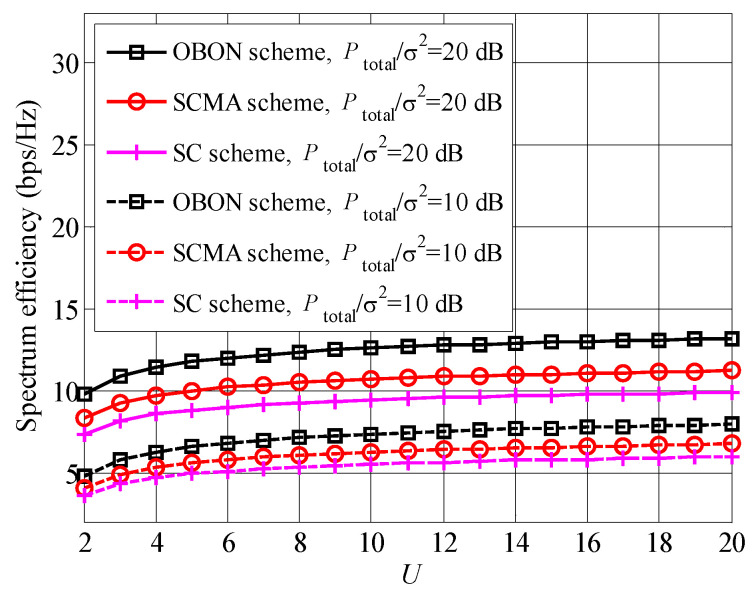
Spectrum efficiencies of OBON, SCMA and SC versus the increased number of waiting users.

In Figure 4, spectrum efficiencies of single-subcarrier (SC), OBON and sparse code multiple-access (SCMA) schemes are compared with the increased number of waiting users *U*. From Figure 4, we can draw that all curves raise with the increased *U*. A lager number of waiting users improves both the multiuser diversity and user pairing gains [25], therefore, a larger *U* leads to higher spectrum efficiency. Comparing the different schemes, the proposed OBON scheme achieves the highest spectrum efficiency, followed by SCMA and SC schemes, since the proposed OBON scheme obtains the largest multiplexing gain and adjusts the wireless resources among the different subcarriers. Obviously, the curves with the larger $\frac{P_{\mathrm{total}}}{\sigma^{2}}$ achieve the higher spectrum efficiencies, no matter of the schemes.

**Figure 5:**
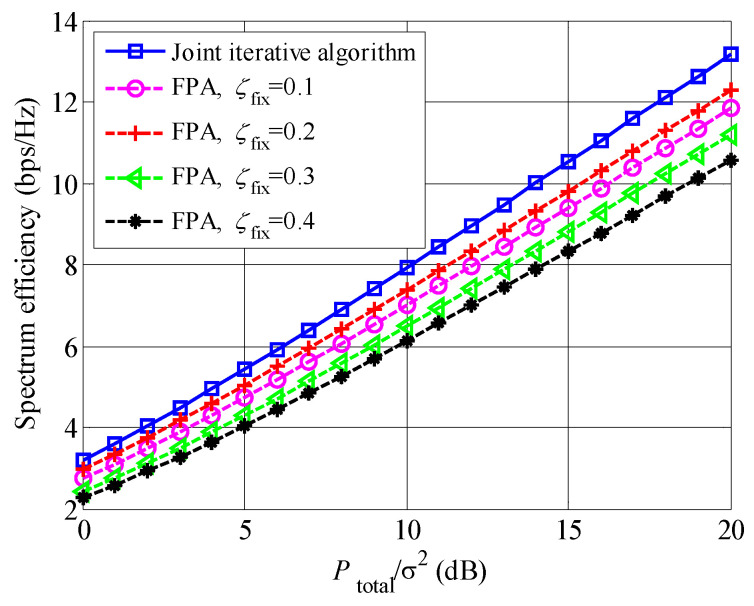
Spectrum efficiency comparison between the proposed joint iterative algorithm and FPA, where U=20.

Figure 5 compares the spectrum efficiencies of our proposed closed-form power allocation (PA) and fixed power allocation (FPA) schemes, where U=20. For the FPA scheme, the exhaustive searching algorithm is also applied to determine subcarrier scheduling and user pairing, and the power of the 1st and 2nd users are respectively $P_{1}=\zeta_{\mathrm{fix}}P_{\mathrm{total}}$, and $P_{2}=\left(1-\zeta_{\mathrm{fix}}\right)P_{\mathrm{total}}$, where $\zeta_{\mathrm{fix}}\in \left(0,0.5\right)$ is a parameter. It is found that the spectrum efficiency of the proposed closed-form PA scheme is always larger than that of the FPA scheme, no matter the values of $\zeta_{\mathrm{fix}}$. The reason is that the proposed closed-form PA scheme can adaptively allocate the power among the users to obtain the maximum spectrum efficiency.

**Figure 6:**
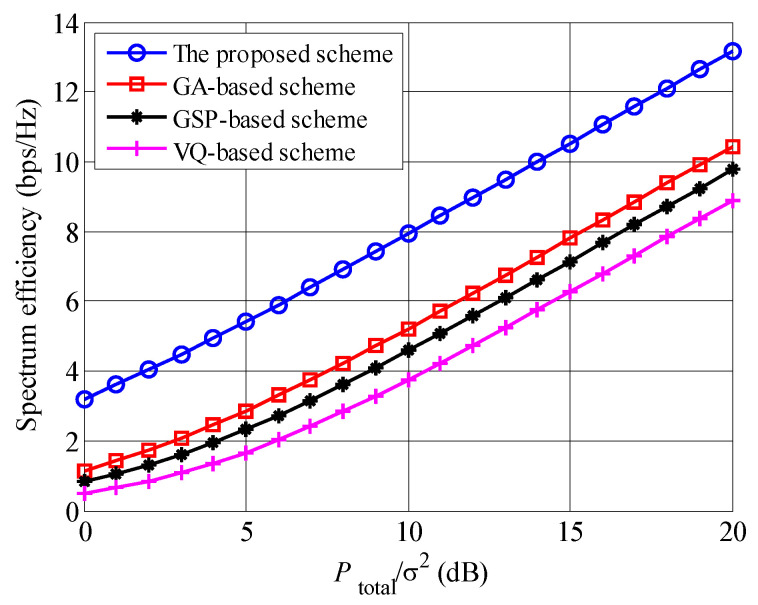
The comparisons of spectrum efficiencies among the proposed, GA, GSP and VQ schemes, where U=20.

In Figure 6 the spectrum efficiencies of the proposed OBON, GSP, GA and VQ scheme are compared under the limited feedback information scenario, where U=20. It is found that our proposed scheme provides the largest spectrum efficiencies, followed by GA, GSP and VQ schemes, no matter of $\frac{P_{\mathrm{total}}}{\sigma^{2}}$. The main reason is that the proposed OBON scheme can provide extra multiuser selection gain, which equals to lnU with many waiting users. Moreover, OBF weights can encourage the fluctuation of equivalent channel and enlarge the equivalent channel coefficients. According to [27,28,29], the beamforming weights of in GA, GSP and VQ schemes are generated through complex matrix calculations, although a random method is applied to obtain the beamforming weights of the proposed scheme. Therefore, the complexity of the proposed scheme is lower than those of GA, GSP and VQ schemes. Comparing the spectrum efficiencies among GA, GSP and VQ schemes, it is seen that the spectrum efficiency of GA scheme is highest, since the distortion of beamforming weights are lowest in GA scheme.

**Figure 7:**
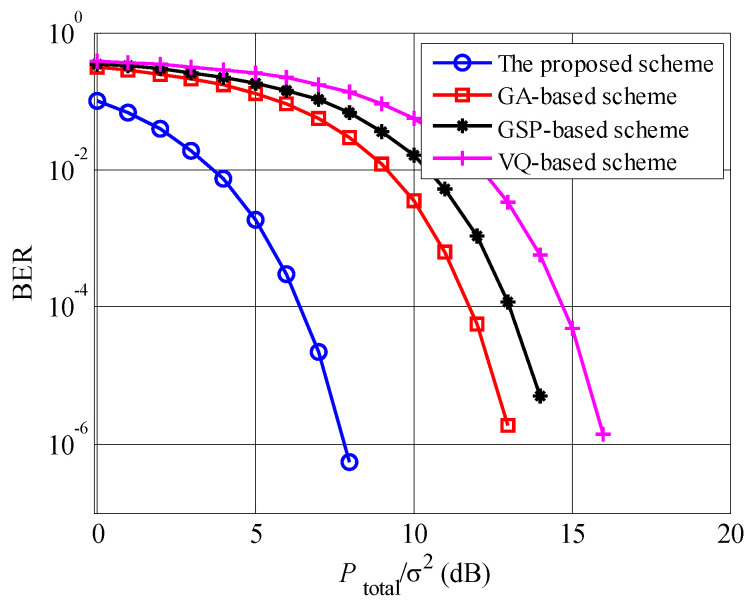
Bit error ratio comparisons among the proposed, GA, GSP and VQ schemes, where U=20.

Figure 7 shows the BER performances of the proposed, GA, GSP and VQ schemes, where U=20. The BER of the proposed scheme is lowest among these schemes, since the proposed scheme obtains both multiuser diversity and beamforming gains. Compared to the GA scheme, the proposed scheme achieves approximately an extra 5 dB gain. According to Figure 6 and Figure 7, the proposed scheme achieves both the highest spectrum efficiency and BER performances among these beamforming schemes with the limited feedback information.

**Figure 8:**
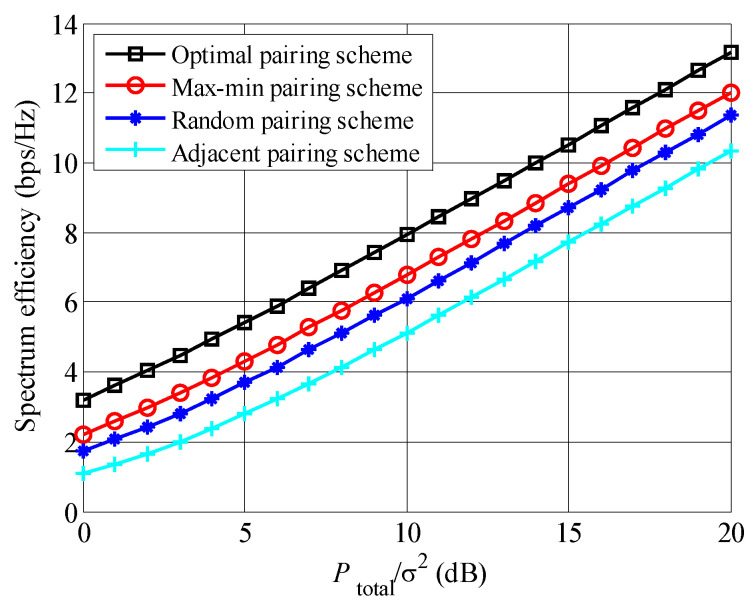
The influences of different user pairing schemes on spectrum efficiencies, where U=20.

In Figure 8, the influences of different user pairing schemes on spectrum efficiencies are indicated, where U=20. “Max-min pairing scheme" means that the users with the maximum and minimum channel qualities are paired. “Random pairing scheme” indicates that the users are randomly paired. In “adjacent pairing scheme”, the BS combines the users with the adjacent channel coefficients to transmit. From Figure 8, it is seen that the proposed optimal scheme achieves the maximum spectrum efficiency, followed by the max-min pairing, random pairing and adjacent pairing schemes. The optimal scheme adaptively arranges the user pairing combination according to the channel coefficients of the users, leading to the largest spectrum efficiency. Comparing the max-min pairing, random pairing and adjacent pairing schemes, the spectrum efficiency of the max-min pairing scheme is highest, and the adjacent pairing scheme provides the lowest spectrum efficiency, which have been analyzed and verified in [32].

**Figure 9:**
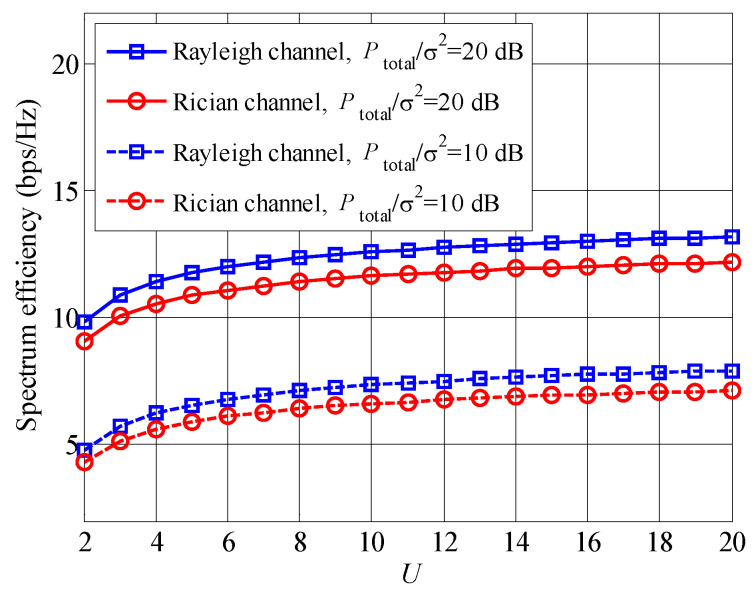
Spectrum efficiency comparison between Rayleigh and Rician fading channels.

The comparison of spectrum efficiencies between Rayleigh and Rician fading channels is shown in Figure 9, where the ratio of direct component and scattering component is set to be 10 in Rician case. It is found that the Rayleigh case always achieves the higher spectrum efficiency than the Rician cases, no matter of $\frac{P_{\mathrm{total}}}{\sigma^{2}}$ and *U*. The reason is that the channel fluctuation of the Rayleigh case is larger than that of the Rician case, leading to a higher multiuser diversity gain. Moreover, all curves raise with the increased $\frac{P_{\mathrm{total}}}{\sigma^{2}}$ and *U*.

**Figure 10:**
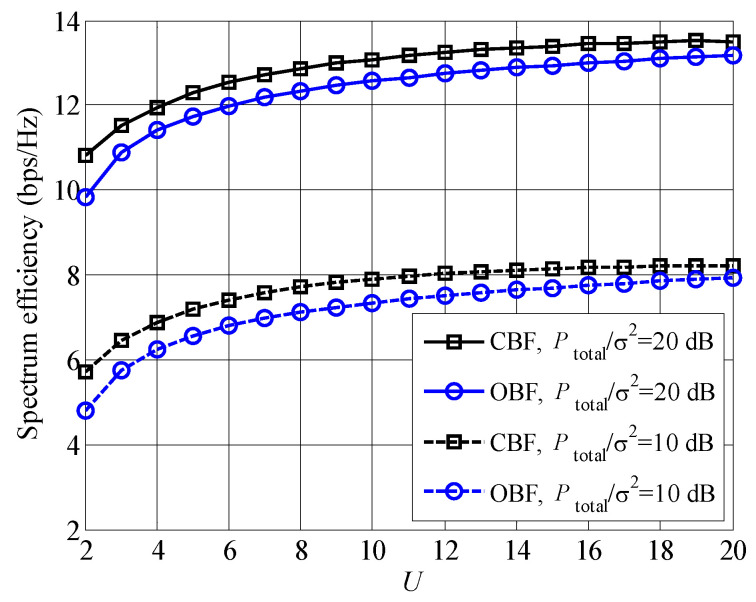
The comparison of spectrum efficiencies between CBF and OBF versus the increased number of waiting users.

In Figure 10, we compare the OBF scheme with CBF, where the joint iterative algorithm is also used to maximize the spectrum efficiency. It is found that CBF scheme provides larger spectrum efficiencies than OBF scheme. However, the complexity of CBF scheme is higher than that of OBF scheme, since the beamforming weights are generated according to the perfect CSI in CBF scheme. According to [31], the computational complexity of CBF weights is $O(N_{T}^{3}\times U^{3})$; on contrast, the computational complexity is about $O(N_{T}\times U)$ of OBF scheme. Compared to CBF scheme, the computational complexity is evidently reduced in OBF scheme, especially with the large numbers of antennas and users. Therefore, these is a trade-off between low complexity and high spectrum efficiency.

**Figure 11:**
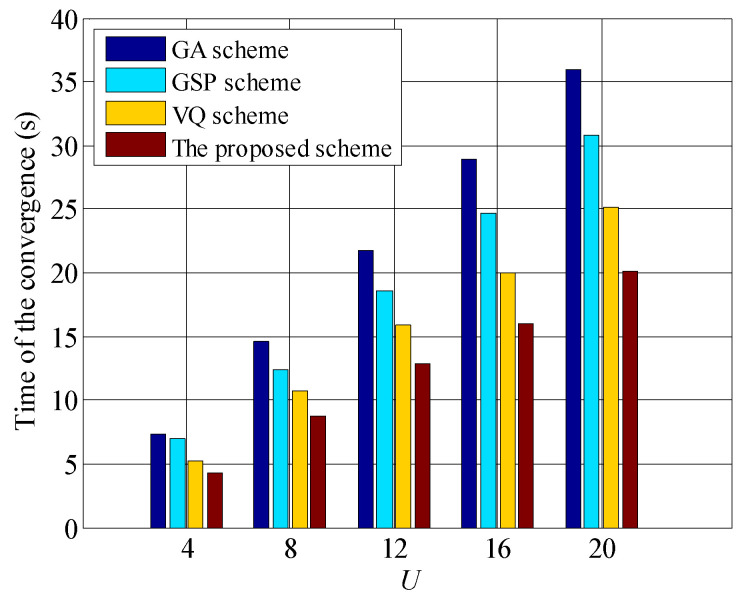
Time of convergence for GA, GSP, VQ and the proposed schemes, where $\frac{P_{\mathrm{total}}}{\sigma^{2}}=10$ dB.

We present the time of convergence for GA, GSP, VQ and the proposed schemes in Figure 11, it is found that the time of all schemes grows with the increased waiting users, and the time of the proposed scheme is lowest. For example, when U=20, the time of the proposed scheme approximates 20 (s), and the GA scheme achieves approximately 36 (s). Moreover, with the increasing of *U*, the gaps between the proposed scheme and other scheme raise, which validates our previous analyses.

**Figure 12:**
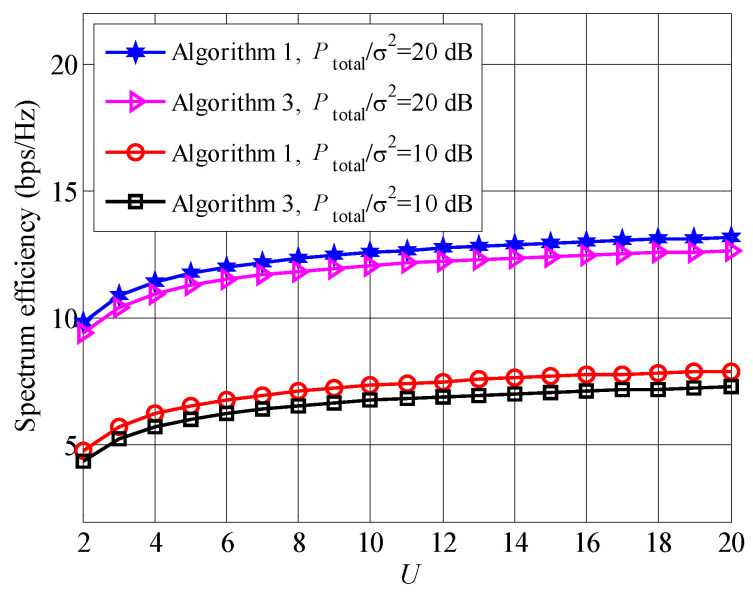
Spectrum efficiency comparison between Algorithms 1 and 3.

To compare the spectrum efficiency between Algorithms 1 and 3 visually, Figure 12 is presented. It is found that the spectrum efficiency of Algorithm 1 is higher than that of Algorithm 3. The reason is that the applied bipartite graph and distributed queue are suboptimal solutions of subcarrier scheduling and user pairing. Although there is a gap between Algorithms 1 and 3, the complexity of Algorithm 3 is lower than that of Algorithm 1. Therefore, Algorithm 3 is a feasible solution of the original resource allocation problem, which is a trade-off between complexity and spectrum efficiency.

## 6. Conclusions

In this paper, a downlink multiuser OBON system is proposed, where multiple users are non-orthogonally multiplexed on different subcarriers with an OBF method. Compared to the conventional multiple-access systems, the spectrum efficiency of OBON is higher, due to the multiuser diversity and multiplexing gains. Then, we formulate an optimization problem to maximize the spectrum efficiency with the QoS constrains of the users. However, the optimization problem is a non-convex problem, which is difficult to solve. To obtain the solution, the proposed optimization problem is divided into two suboptimal problems, and an iterative algorithm is applied to jointly consider both the suboptimal problem. Finally, numerical results are presented. Compared to other beamforming schemes with low feedback information, it is found that the proposed system can obtain higher spectrum efficiency with lower computation complexity. Moreover, there are still many interesting topics left in OBON systems, i.e., peak to average power ratio (PAPR) problem, multiplexing more users on each subcarrier and the performance loss caused by correlated channels.

## Figures and Tables

**Table 1:** The simulation parameters.

Simulation Parameters	Value
Number of transmit antennas $N_{T}$	2
AWGN variance $\sigma_{z_{u}}^{2}$	$\sigma^{2}$
Ratio of transmit power to noise variance $\frac{P_{\mathrm{total}}}{\sigma^{2}}$	[0–20] (dB)
Number of subcarriers $N_{c}$	64
Number of users *U*	[1–20]

## Data Availability

Not applicable.

## Appendix A

The notations of this paper are shown in Table A1.

**Table A1:** The meanings of the notations.

Notation	Meaning	Notation	Meaning
$B_{sub}$	Bandwidth of each subcarrier	*D*	The suboptimal issue
$d_{n_{c}}$	The superposition transmit signal	$\phi_{n_{t}}$	The phase of $w_{n_{t}}$
$E_{\mathrm{total}}$	Total SE	$e_{k_{n_{c}}}$	The minimum SE of each user
$F_{D}$	DFT matrix	$F_{I}$	IDFT matrix
G	The feasible interval	$G_{\zeta ,\mathrm{left}}$	The left end of G
$G_{\zeta ,\mathrm{right}}$	The right end of G	$h_{u}$	Vector form of channel
$h_{n_{t},u}$	Channel coefficient	*i*	Factor in the proposed algorithm
*j*	The symbol of imaginary number	$l_{n_{c},u}$	Channel coefficient of subcarrier
$N_{T}$	The number of transmit antennas	$N_{c}$	The number of subcarriers
$y_{u}$	The received signals of users	*K*	The number of multiplexing users
$w_{n_{t}}$	The random coefficient	$\sigma^{2}$	Variance
κ	The mapping between $k_{n_{c}}$ and *u*	$s_{p}$	The transmit signal of pilot
$\lambda_{p}$	Complexity of one derivation	$P_{\Delta }$	The minimum difference of power
$n_{t}$	The $n_{t}$th transmit antenna	$h_{u}^{\mathrm{equ}}$	Equivalent channel
$R_{k_{n_{c}}}$	Rate	$U_{s}$	User set
$z_{u}$, ${\tilde{z}}_{u}$	AWGN vector	$z_{u}$	AWGN
w	The vector form of $w_{n_{t}}$	ζ	Power factor
$y_{p}$	The received signal of pilot	${\tilde{y}}_{p}$	DFT of $y_{p}$
${\tilde{h}}_{u}^{\mathrm{equ}}$	DFT of $h_{u}^{\mathrm{equ}}$	$P_{\mathrm{total}}$	The total power
$x_{p}$	Pilot signal	$x_{p}$	The vector form of pilot
${\bar{x}}_{p}$	IDFT of $x_{p}$	β	Factor of IDFT
$\sqrt{\alpha_{n_{t}}}$	The amplitude of $w_{n_{t}}$	*U*	The number of waiting users
$k_{n_{c}}$	The $k_{n_{c}}$th user	κ	The mapping between $k_{n_{c}}$ and *u*
*u*	The *u*th user	ϑ	Factor in the proposed algorithm
$P_{k_{n_{c}}}$	The allocated power	$x_{k_{n_{c}}}$	User’s signal
$y_{k_{n_{c}}}$	The received signal of user	$\gamma_{k_{n_{c}}}$	SINR
$M_{\mathrm{low}}$	The parameter in Algorithm 3	ς	The parameter in Algorithm 3
